# Optimizing Tourism Routes: A Quantum Approach to the Profitable Tour Problem

**DOI:** 10.3390/e28020153

**Published:** 2026-01-29

**Authors:** Xiao-Shuang Cheng, You-Hang Liu, Xiao-Hong Dong, Yan Wang

**Affiliations:** 1 School of Geography and Tourism, Qilu Normal University, Jinan 250000, China; 20159476@qlnu.edu.cn (X.-H.D.); wangyan5106@163.com (Y.W.); 2 School of Computer Science and Artificial Intelligence, Aerospace Information Technology University, Jinan 250000, China; outsider_hang@163.com

**Keywords:** quantum computing, tourism management, profitable tour problem, variational quantum eigensolver

## Abstract

The Profitable Tour Problem is a well-known NP-hard optimization challenge central to tourism planning, aiming to maximize collected profit while minimizing travel costs. While classical heuristics provide approximate solutions, they often struggle with finding globally optimal routes. This paper explores the application of near-term quantum computing to this problem. We propose a framework based on the Variational Quantum Eigensolver to find high-quality solutions for the Profitable Tour Problem. The core of our contribution is a novel methodology for constructing a constraint-aware variational ansatz that directly encodes the problem’s hard constraints. This approach circumvents the need for large penalty terms in the Hamiltonian problem, which are often a source of optimization challenges. We validate our method through numerical simulations on a representative tourism scenario of up to 25 qubits. The results demonstrate the viability of the approach, achieving high solution accuracy consistent with brute-force enumeration for smaller instances. This work serves as a proof-of-concept for applying Variational Quantum Eigensolver to complex tourism optimization problems and provides a basis for future exploration on real quantum hardware.

## 1. Introduction

The tourism industry is a significant driver of the global economy, where operational efficiency in resource allocation, scheduling, and routing is paramount. A canonical challenge in this domain is the Profitable Tour Problem (PTP) [1], a variant of the classic Traveling Salesman Problem (TSP). The PTP seeks to determine a tour that maximizes the total profit collected from a subset of visited locations while accounting for the travel costs between them. Due to its NP-hard nature, finding exact optimal solutions for large-scale PTP instances is computationally intractable for classical computers. Consequently, research has predominantly focused on classical heuristics like genetic algorithms or simulated annealing, which find approximate solutions but do not guarantee optimality and can get trapped in local minima [2,3,4].

The advent of quantum computing offers a new paradigm for tackling such intractable optimization problems [5,6,7,8,9,10]. By leveraging principles like superposition and entanglement, quantum algorithms promise significant speedups for certain computational tasks. In the current era of Noisy Intermediate-Scale Quantum (NISQ) devices [11], hybrid quantum-classical algorithms have emerged as a particularly promising avenue. The Variational Quantum Eigensolver (VQE) is one such algorithm [12], designed to find the ground state of a given Hamiltonian, which can be mapped to the solution of an optimization problem. VQE has been explored for applications in quantum chemistry, finance, and logistics [13,14,15], but its application to tourism management remains nascent.

In this research, we introduce a method to solve the PTP using VQE. Our primary contribution is the adaptation of a constraint-preserving ansatz, inspired by recent work in quantum optimization [16], to the specific structure of the PTP. This involves designing a problem-specific Hamiltonian and a variational quantum circuit that generates only valid tour routes, thereby simplifying the optimization landscape.

The remainder of this paper is organized as follows. Section 2 provides the necessary preliminaries on the VQE algorithm. Section 3 details our methodology, including the Quadratic Unconstrained Binary Optimization (QUBO) formulation of the PTP and the construction of our constraint-aware ansatz. Section 4 presents a numerical simulation to validate the algorithm and analyzes its complexity. Section 5 discusses the limitations of this study, directions for future work, and concludes the paper.

## 2. Materials and Methods

VQE is a hybrid quantum-classical algorithm designed to find the lowest eigenvalue of a Hamiltonian, H. This is equivalent to finding the ground state $\left|\Psi_{0}\right\rangle$ that minimizes the expectation value ⟨Ψ(θ)|H|Ψ(θ)⟩. The process is particularly suited for optimization problems, where the problem can be mapped to a Hamiltonian whose ground state corresponds to the optimal solution.

**Figure 1:**
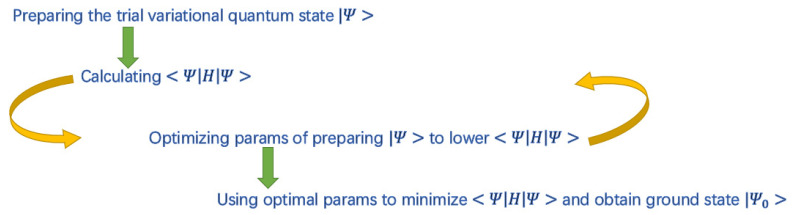
The sketch of VQE for searching the ground state of Hamiltonian H.

The VQE algorithm operates in a loop, as shown in Figure 1.

State Preparation: A parameterized quantum circuit, known as a variational ansatz U(θ), is used to prepare a trial quantum state |ψ(θ)⟩=U(θ)|0⟩ from an initial state |0⟩. The parameters θ are adjustable classical variables.

Measurement: The expectation value of the Hamiltonian, ⟨H⟩=⟨ψ(θ)|H|ψ(θ)⟩, is measured on a quantum computer. This step often involves decomposing H into a sum of simpler, measurable terms and averaging the results over many measurements.

Classical Optimization: The measured expectation value is passed to a classical optimizer. The optimizer suggests a new set of parameters, intended to lower the expectation value.

Iteration: The above steps are repeated until the expectation value converges to a minimum. The resulting state $\left|\psi (\theta_{optimal})\right\rangle$ is the algorithm’s approximation of the ground state, and from it, the solution to the optimization problem can be extracted.

## 3. Methodology: A VQE Framework for the PTP

Our approach involves translating the PTP into a QUBO formulation, mapping it onto a Hamiltonian, and then using VQE with a specially designed ansatz to find the ground state.

### 3.1. QUBO Formulation of the PTP

**Figure 2:**
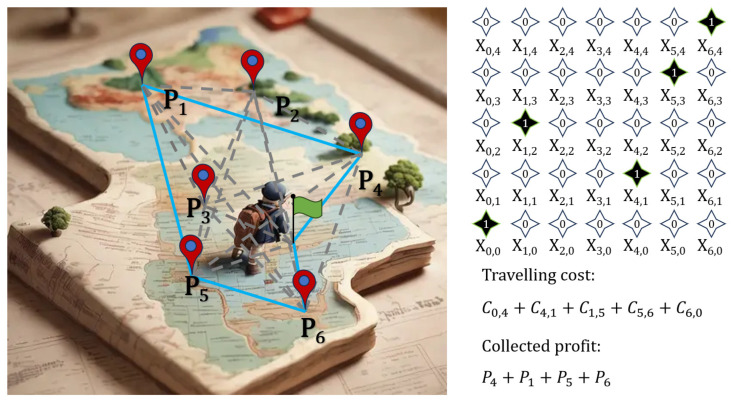
A diagram of PTP with six possible destinations. Assuming that a traveler will travel to four spots, an example of route encoding is illustrated on the right, which indicates a path order of starting point→4→1→5→6→starting point, highlighted with the blue line. All possible routes are illustrated with gray dashed lines. Traveling cost and collected profit are also presented.

The PTP can be formulated on an undirected graph G=(V, E), where V is the set of n+1 vertices (a starting point and n destinations) and E is the set of edges. Each vertex i ∈ {0, …, n} has an associated profit $P_{i}$, and each edge (i, j) has a travel cost $C_{ij}$. We use a binary variable, $x_{ik}$, to indicate whether or not the traveler is at the $i_{th}$ destination (i=0 denotes the point of departure) at the $k_{th}$ step in the tour route (k=0 denotes the departure time), as shown in the example illustration (Figure 2).

The objective is to minimize the total cost function

(1) $$Cost\;function=\sum_{k=0}^{d}\sum_{i=0}^{n}\sum_{j=0}^{n}C_{i,j}x_{i,k}x_{j,k+1}-\sum_{k=1}^{d}\sum_{i=1}^{n}P_{i}x_{i,k}$$

Where d indicates the tour length among n possible destinations.

The cost function is subject to two main constraints.

Constraint 1. The traveler must be at exactly one location at each step: $\sum_{i=0}^{n}x_{i,k}=1,\;k=0,1,\ldots ,d$.

Constraint 2. Each destination is visited at most once: $\sum_{k=0}^{d}x_{i,k}=0\;or\;1,\;i=0,1,\ldots ,n$.

### 3.2. QUBO Transformation and Hamiltonian Mapping

To solve this optimization problem with VQE, we must first map the classical cost function (Equation (1)), which is expressed in terms of binary variables $x_{i,k}\in \{0,1\}$, into a quantum Hamiltonian whose ground state corresponds to the optimal solution. This is achieved by replacing each binary variable $x_{i,k}$ with a corresponding quantum operator. The standard mapping for this transformation is $x_{i,k}=\frac{I-\sigma_{z}^{i,k}}{2}$, where I is the identity operator and $\sigma_{z}^{i,k}$ is the Pauli-Z operator acting on the qubit corresponding to ${qubit}_{i,k}$. This mapping holds because the operator $\frac{I-\sigma_{z}^{i,k}}{2}$ has eigenvalues of 0 and 1, which correspond to the computational basis states |0⟩ and |1⟩ of the qubit.

By substituting this operator mapping into our original cost function (Equation (1)), the classical objective is transformed into an Ising-type Hamiltonian operator, $H_{problem}$.

(2) $$H_{problem}=\sum_{k=0}^{d}\sum_{i=0}^{n}\sum_{\;j=0}^{n}C_{i,j}\frac{I-\sigma_{z}^{i,k}}{2}\frac{I-\sigma_{z}^{j,k+1}}{2}-\sum_{k=1}^{d}\sum_{i=1}^{n}P_{i}\frac{I-\sigma_{z}^{i,k}}{2}$$

This formulation captures the correlations between variables as two-qubit $\sigma_{z}^{i,k}\sigma_{z}^{j,k+1}$ interaction terms, which is crucial for leveraging quantum effects. The task for the VQE algorithm is then to find the ground state of this specific $H_{problem}$ by minimizing its expectation value ${\langle H}_{problem}\rangle$.

A standard method to enforce constraints is to add a penalty term $H_{penalty}$ to the Hamiltonian $H\;=\;H_{problem}+A\times H_{penalty}$, which increases the energy for states that violate the constraints. However, this approach has several drawbacks. It introduces an additional hyperparameter that requires tuning, increases the complexity of the Hamiltonian, and, most critically, does not restrict the search to the space of valid solutions. Searching over a large space of invalid states can lead to the “Barren Plateau” phenomenon, where gradients vanish exponentially, making training intractable.

Therefore, a key point of our work is to construct trial quantum states that inherently satisfy the problem’s constraints. Our method iteratively builds the d×n matrix of qubits representing the tour. This strategy, which avoids the need for penalty terms, is crucial for reducing the search space and mitigating issues like Barren Plateaus.

The core of our circuit design is an adaptation and extension of the work by [16], who first proposed this problem-specific approach for the standard TSP. Our work builds upon their foundational concept in two significant ways. Firstly, we apply this methodology to the PTP, a more complex variant where the objective is to select a subset of destinations that maximizes net profit. This required us to formulate a novel objective Hamiltonian specific to PTP. Secondly, we adapt their circuit construction technique, to generate trial states that specifically satisfy the unique constraints of our PTP formulation by constructing a valid tour matrix of dimension d×n through an iterative process. Therefore, while the underlying principle of a constraint-preserving ansatz is inspired by [16], its application to PTP, the corresponding Hamiltonian formulation, and the specific circuit implementation for the subset tour destinations represent the novel contributions of this study.

### 3.3. Constraint-Aware Variational Ansatz

**Figure 3:**
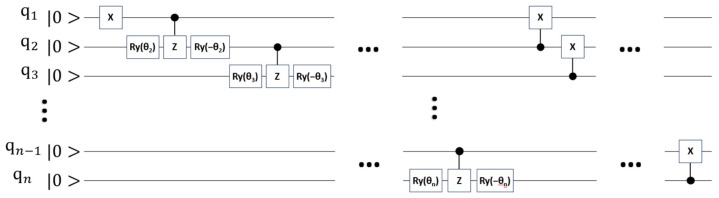
The quantum circuit for preparing variational W−state of n qubits.

An essential part of our methodology is the construction of a variational ansatz that inherently satisfies the problem’s hard constraints. A key component of our iterative ansatz construction is a module that prepares a variational W−state [17]. It is important to clarify that this module is not the final ansatz itself, but serves as a fundamental building block used to coherently select one location at each step of the tour. Variational W−state is such a superposition whose components are quantum states with one qubit at |1⟩ and others at |0…0⟩. The construction of quantum circuit for generating variational W−state of n qubits is illustrated in Figure 3.

The output of the circuit is $\left|{Wstate}_{n}\rangle =a_{1}\right|10\ldots 00\rangle +a_{2}\left|01\ldots 00\rangle +\cdots +a_{n-1}\right|00\ldots 10\rangle +a_{n}|00\ldots 01\rangle$, where the coefficients depend on the parameters $\left\{\theta_{2},\;\theta_{3},\ldots ,\theta_{n}\right\}$ in Figure 3. It can be shown that the coefficient $a_{i}$ is the trigonometric function of the whole or part of the parameters $\left\{\theta_{2},\;\theta_{3},\ldots ,\theta_{n}\right\}$. This dependence implies that the quantum superposition can be steered towards a specific binary string by adjusting these parameters.

**Figure 4:**
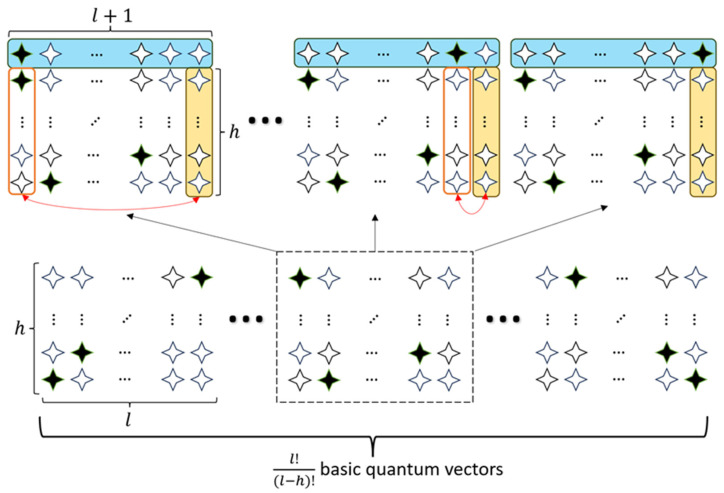
An illustration of expanding the dimension of trial matrices from h×l to (h+1)×(l+1). In this schematic, the blue bar represents the newly added row prepared in the W−state, the yellow bar represents the newly added column of |0…0⟩, and the black marker indicates the |1⟩ component within that state. The red boxes represent CSWAP operations which are controlled by the W−state and therefore act below the black box in the W−state.

**Figure 5:**
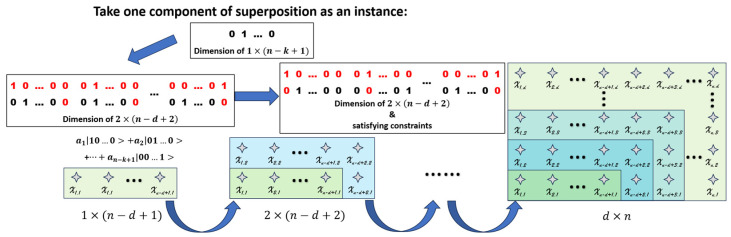
A sketch of iterative generation of trial matrices with dimension of d×n. The gray stars represent the uncertain value of qubits. That is to say, every matrix represents a quantum superposition. An example of matrix extension is also given on up left.

Apparently, the basic components of W−state can only satisfy one of the constraints. With an attempt to construct the trial quantum states satisfying both, a carefully designed component is utilized. The full ansatz is constructed through d iterative steps, depicted schematically in Figure 4 and Figure 5. To formalize the core mechanism, the detailed unitary operations for a single iterative step (expanding an h×l valid tour to an (h+1)×(l+1) tour) are given as follows.

Assuming that we already have a quantum superposition that forms the solution matrix with the dimension of h×l, we can expand the dimension of the trial quantum states to (h+1)×(l+1) by adding a new row composed of the variational W−state of l+1 qubits, and a new column composed of $\left|\underset{h\;qubits}{\underbrace{0\ldots 0}}\right\rangle$, as shown in Figure 4. Formally, this corresponds to preparing an expanded state of the form $|\varphi_{before}\rangle ⨂|W_{l+1}\rangle ⨂{\left|0\ldots 0\right\rangle }_{h}$, where $\left|\varphi_{before}\right\rangle$ is a basis state of a valid h×l tour matrix.

However, the expanded trial quantum superpositions are not guaranteed to meet the constraints. Subsequently, h×l Controlled−SWAP (CSWAP) gates are used to swap the new column with each column in the old matrix under the control of the elements in the newly added row. The CSWAP gate is a three-qubit unitary operator defined by its action $\{\begin{array}{c} CSWAP{\left|0\right\rangle }_{control}{\left|\varphi_{1}\right\rangle }_{target}{\left|\varphi_{2}\right\rangle }_{target}\;=\;|0\rangle |\varphi_{1}\rangle |\varphi_{2}\rangle \\ CSWAP{\left|1\right\rangle }_{control}{\left|\varphi_{1}\right\rangle }_{target}{\left|\varphi_{2}\right\rangle }_{target}\;=\;|1\rangle |\varphi_{2}\rangle |\varphi_{1}\rangle \end{array}$. This use of controlled operations is crucial, as it creates entanglement between the rows of the tour matrix to ensure the constraint 2 is satisfied. The resulting ansatz is therefore not a simple product of W−states, but a more complex, entangled superposition over the space of valid solutions.

The example sketched in Figure 4 illustrates that the application of CSWAP gates will swap the $i_{th}$ column with the newly added $\left|\underset{h\;qubits}{\underbrace{0\ldots 0}}\right\rangle$ only if the $i_{th}$ element of the added variational W−state is |1⟩. By applying these unitary operators, with the qubits of the $\left|W_{l+1}\right\rangle$ state acting as controls, we perform the column swapping depicted in Figure 4, ensuring the constraints of the tour are maintained.

With the above approach, we can obtain the trial matrices of higher dimension from the lower ones that satisfy the constraints. Therefore, starting from the quantum states that both are easy to be prepared and satisfy the constraints, the trial matrices with dimension of d×n can be generated iteratively.

Step 1, prepare the variational W−state of n−d+1 qubits, which forms a matrix with the dimension of 1×(n−d+1). This quantum state with only one row satisfies the both constraints.

Step 2, add a new row composed of the variational W−state and a new column composed of |0…0⟩.

Step 3, swap the new column with every column in the old matrices under the control of the corresponding element in the added row.

Step 4, repeat Step 2 and Step 3 iteratively, until the number of rows increases from 1 to d and the number of columns increases from (n−d+1) to n.

An illustration of the iterative generation of the trial matrix with dimension of d×n is presented in Figure 5. Taking one component of the superposition prepared in step 1 as an example, it is clear that after extending the matrix and applying CSWAP gates, the newly generated matrices have only one qubit at |1⟩ per row, and one or none qubit at |1⟩ per column.

This iterative process starts with a 1×(n−d+1) matrix and expands it to the final d×n matrix. The resulting quantum state is a superposition of only valid tour routes, with amplitudes controlled by the variational parameters. This construction can also be linked to “warm-starting” techniques. If a good classical solution is known, its structure could be used to set the initial variational parameters, potentially accelerating convergence. For the numerical simulations presented in this work, we specify the initial state by setting all variational parameters to 1, which corresponds to preparing a superposition over all valid tour routes as the starting point for the optimization.

### 3.4. Training Method

We have constructed the trial quantum states that satisfy the constraints and cover all possible tour routes. The ability to process all possible solutions simultaneously comes at a cost of polynomial complexity both in the space and computation (as shown in Table 1). However, this is still an acceptable sacrifice compared with exponential scaling of classical methods.

Before proceeding to the numerical simulation, it is useful to analyze the resources required by our proposed method, as summarized in Table 1.

The number of variational parameters, nd−d22−d/2, originates from our iterative ansatz construction. As described in Section 3.3, the circuit is built in d steps. At each step k, we introduce a variational W−state on n−d+k qubits, which requires n−d+k−1 variational parameters. Summing these parameters over all d steps, $\sum_{k=1}^{d}\left(n-d+k-1\right)$, yields the total count.

Similarly, the number of Hamiltonian terms to be evaluated, $n^{2}d\;-\;n^{2}\;+\;n$, is determined by expanding the final objective Hamiltonian after its mapping to Pauli operators and dropping the constant terms. The objective Hamiltonian H is ultimately written as

(3) $$H=\sum_{k=1}^{d-1}\sum_{i,\;j=1,\;i\neq j}^{n}\frac{C_{i,j}}{4}\sigma_{z}^{i,k}\sigma_{z}^{j,k+1}-\sum_{k=2}^{d-1}\sum_{i,\;j=1,\;i\neq j}^{n}\left(\frac{C_{i,j}}{2}-\frac{P_{i}}{2}\right)\sigma_{z}^{i,k}-\;\sum_{i,\;j=1,\;i\neq j}^{n}\left(\frac{C_{i,j}}{4}+\frac{C_{i,0}}{2}-\frac{P_{i}}{2}\right)\sigma_{z}^{i,1}-\sum_{i,\;j=1,\;i\neq j}^{n}\left(\frac{C_{i,j}}{4}+\frac{C_{i,0}}{2}-\frac{P_{i}}{2}\right)\sigma_{z}^{j,d}\;$$

Please note that both the costs from the point of departure to the first destination and from the last destination to the point of departure are also included in (3) as the point of departure is denoted as the $0_{th}$ destination.

The number of Hamiltonian terms includes all unique single-body (Pauli-Z) and two-body (Pauli-ZZ) interaction terms that must be measured to calculate the objective function’s expectation value. This formula provides an upper bound on the terms requiring measurement.

In general, the optimization of the objective Hamiltonian necessitates the approximate evaluation of the gradient, which is the function of parameters. The standard calculation of the gradient in variational quantum circuits utilizes the parameter-shift rule [18]. This method requires the repeated application of the same variational quantum circuit with variational parameters. Due to the high time cost associated with a large number of parameters, the training phase has become a significant bottleneck of the practical deployment of large-scale variational quantum models. Various strategies have been developed such as the simultaneous permutation stochastic approximation method [19], or calculating the numerical gradient of multivariate functions with only one query of the quantum computer [20]. However, this is still an open question and beyond the scope of this study. With the purpose of validating our method, it is reasonable to assume that the ground state can be found with the achievable iterations.

## 4. Numerical Simulation

### 4.1. Experimental Setup

To validate the proposed method, we present a proof-of-principle numerical simulation using PyqPanda [21]. The use of a classical simulator is a necessary expedient due to the limited availability of large-scale quantum hardware.

Our analysis begins with a specific problem instance. Considering a scenario where a traveler plans to visit d=3 destinations out of n=5 possibilities, the cost and profit parameters for this instance, which will be used for our primary analysis, are detailed in Table 2. The cost $C_{i,j}$ is the Euclidean distance between destinations. While this is a simplification, other factors like traffic and transportation mode can be modeled as scaling factors without changing the algorithm’s validity. The profit $P_{i}$ for each destination is calculated as a weighted average of several factors, as detailed in Table 2. For inverse indicators such as Entrance Ticket Price and Crowd Density, a higher raw value corresponds to a lower effective profit. This is handled by the weighting scheme, in which such factors make a negative contribution to the final score.

The weighted average profit $P_{i}$ of the $i_{th}$ destination is calculated with

(4) $$P_{i}=\sum_{j}W_{j}P_{j}\;$$

(5) $$s.t.\;\sum_{j}\left|W_{j}\right|=1\;$$

The traveling cost from destination i to j is calculated with

(6) $$C_{i,j}=\sqrt{{\left(D_{i}\left[0\right]-D_{j}\left[0\right]\right)}^{2}+{\left(D_{i}\left[1\right]-D_{j}\left[1\right]\right)}^{2}}\;$$

Where D is the Cartesian coordinates.

### 4.2. Results and Analysis

**Figure 6:**
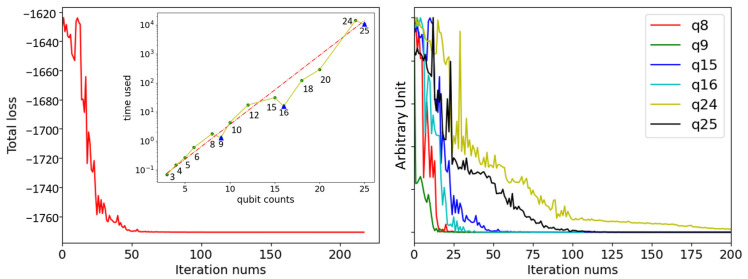
**Left**: The loss curve for the scenario with 3 destinations in 5 possible spots. The inset illustrates the exponential increase in simulation time (in seconds) with the number of qubits. Blue triangles represent the notable dips, and the dashed red line indicates the fit curve. **Right**: The loss curves for training the PTP model of 8, 9, 15, 16, 24 and 25 qubits. The faster convergence for 9, 16 and 25 qubits can be attributed to a smaller search space of valid routes.

As listed in Table 1, there are nd−d22−d/2 parameters to be optimized and $n^{2}d-n^{2}+n$ Hamiltonian terms to be evaluated. With initializing all parameters to 1, a quantum superposition containing all potential tour routes is prepared and the objective Hamiltonian has the initial value of −1630.29. By training the parameters to lower the objective Hamiltonian, the minimized value of −1770.81 is obtained after around 60 iterations, as plotted in Figure 6. Finally, the best tour route is starting point→5→4→1→starting point, which is consistent with the result obtained by classical enumeration.

To further evaluate the performance of our VQE framework in terms of solution quality, we benchmarked it against a standard classical algorithm. For this classical baseline, we implemented a simple greedy search algorithm, which constructs a tour by iteratively selecting the unvisited destination with the highest profit-to-cost ratio from its current location. A statistical analysis was then performed by testing this greedy algorithm on 100 randomly generated instances for each (n, d) combination. The success rate of the greedy algorithm is summarized in Table 3. The results reveal that the probability of the greedy heuristic finding the optimal solution drops dramatically as the problem complexity increases. This systematically demonstrates the inherent limitation of such locally optimal methods when navigating a complex combinatorial solution space.

**Figure 7:**
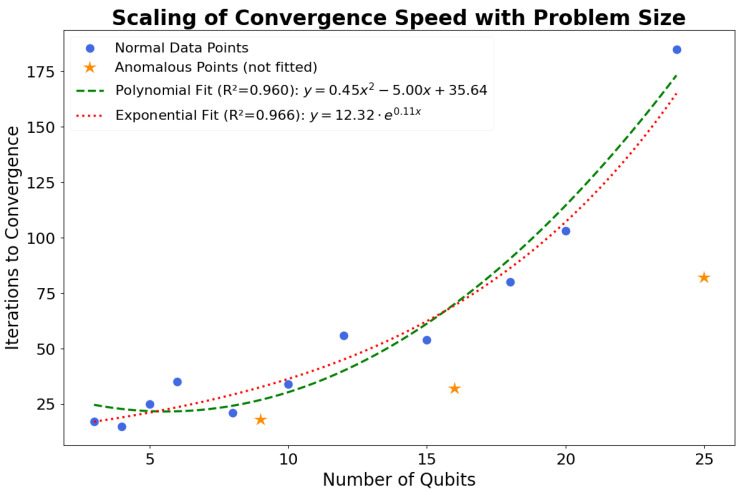
Scaling of the number of iterations required for convergence as a function of problem size. The blue circles represent the data points for typical instances, which were used for curve fitting. The orange stars highlight the anomalous instances (9, 16, and 25 qubits), which converge faster than the general trend and were excluded from the fitting process. A second-degree polynomial (green dashed line) and an exponential function (red dotted line) were fitted to the normal data points. Both models show a strong correlation, with R-squared values of 0.960 and 0.966, respectively, indicating that the exponential model provides a marginally better fit for the general trend.

In stark contrast, our VQE framework demonstrated much higher reliability. To manage the high computational cost of quantum simulation, we performed a focused validation by applying our VQE algorithm to 10 randomly generated instances for each of the problem sizes that were also analyzed for convergence speed (i.e., the qubit counts shown in Figure 6 and Figure 7). For all of these tested instances, the VQE framework successfully converged to the true optimal solution, which was determined by a brute-force search. It is important to note that these VQE tests were limited to problem sizes up to 25 qubits due to the capacity of our classical simulator. Even within this scope, the comparison highlights a key potential advantage of our quantum approach. A more reliable ability to find globally optimal solutions where simple, fast classical heuristics are prone to fail.

Beyond solution quality, another crucial aspect of the algorithm’s performance is its scalability. We simulated several problem sizes, defined by (n, d) pairs which correspond to the different qubit counts in Figure 6 (e.g., (n=4, d=2) for 8 qubits, (n=3, d=3) for 9 qubits, and so on). To test the robustness of our method, for each problem size, we generated 10 distinct random instances by varying destination coordinates and profits. The VQE algorithm was then applied to each of these test cases.

The execution time for each simulation was recorded to analyze how the time scales with the problem size. As shown in the inset of Figure 6, the time consumed exhibits an exponential scale with the increasing qubit counts, appearing as a linear trend on the log-scale plot. Each point in the curve is the average of ten experiments. However, there are three notable dips in the time consumed for the 9, 16, and 25 qubits instances, contradicting the general trend.

To investigate this anomaly, we examined the training dynamics. The right panel of Figure 6 plots the loss curves for the anomalous instances against their immediate predecessors (e.g., 9 vs. 8 qubits). To facilitate comparison of convergence trends, the loss data for different qubit counts has been normalized, and the *Y*-axis uses arbitrary units. The data reveals that the 9, 16, and 25 qubits instances converge significantly faster. Although a single iteration is more costly for a higher qubit count, the drastic reduction in the required number of iterations leads to a shorter total execution time. We attribute this faster convergence to the problem structure of these specific sizes, which features fewer possible tour routes and fewer variational parameters to optimize, allowing the optimizer to find the solution more efficiently.

To address the scaling of the algorithm’s convergence speed, we analyzed the number of iterations required to reach the final solution as a function of problem size. The results are presented in Figure 7. We first identified the anomalous instances (9, 16, and 25 qubits), which converge significantly faster due to their smaller search space as previously discussed. These points, marked as orange stars, were excluded from the trend analysis to better characterize the general scaling behavior of the algorithm.

We then fitted the remaining normal data points (blue circles) with both a second-degree polynomial model and an exponential model ($y\;=\;ae^{bx}$). Both models describe the data trend well. The polynomial fit yielded an R-squared ($R^{2}$) value of 0.960, while the exponential fit yielded a slightly higher $R^{2}$ of 0.966.

While the exponential model provides a marginally better fit for the tested range, the small difference in R^2^ values suggests that polynomial scaling cannot be conclusively ruled out. Moreover, the small coefficient in the exponent (b ≈0.11) indicates that the growth, while formally exponential, is slow in this regime. This entire analysis highlights a crucial aspect of variational algorithms. While the complexity of preparing the ansatz and measuring the Hamiltonian for a single iteration is polynomial, the overall runtime is also dependent on the number of optimization iterations, which appears to be a non-trivial, potentially exponential function of problem size. This observation aligns with known challenges in the field regarding the trainability and scaling of heuristic quantum algorithms.

Finally, it should be mentioned that the above analysis is wholly based on the classical simulation of quantum computing due to the absence of usable large-scale quantum computers at the current stage. In this condition, the time complexity of simulating the single run of quantum circuit is exponentially scale with the linear increase of qubits. However, as for the real quantum computing device, there are different analysis methods.

### 4.3. Complexity Analysis

The overall runtime complexity of the VQE algorithm is determined by several factors. A simplified model for the total time complexity O(T) can be expressed as: $O(T)\propto (N_{iter}\times N_{terms}\times N_{shots}\times T_{circuit})$, where $N_{iter}$ is the number of optimization iterations to convergence, $N_{terms}$ is the number of terms in the Hamiltonian, $T_{circuit}$ is the execution time of a single circuit run, and $N_{shots}$ is the number of measurements per term. The number of shots, $N_{shots}$, does not depend on the problem size, but rather on the desired precision ε of the expectation value estimation, typically scaling as $O(1/\varepsilon^{2})$ [12].

Our analysis will focus on how these components scale with the problem size. As the number of destinations is generally limited due to objective constraints such as idle time and the traveler’s financial strength, we consider that d is a constant and has no influence on the complexity here. For this work, we consider the primary scaling variable to be n (the number of possible destinations).

For our proposed method, the resources required per iteration scale polynomially with n. This cost is dominated by measuring the $O(n^{2})$ terms of the Hamiltonian, where each measurement requires one execution of the ansatz circuit. The gate complexity of this circuit, $T_{circuit}$, is determined by its construction, which consists of two main operations within each of the d iterative steps:

1. W−state Preparation: This involves creating a variational W−state on a number of qubits scaling linearly with n. As can be derived from the circuit diagram in Figure 3, the total number of quantum gates required for this operation is 4k−3 for k qubits, which is O(n).
2. Controlled−SWAP Application: This involves applying a series of Controlled−SWAP gates. As described in Section 3.3, the number of these gates applied in each of the d steps is also polynomial in n.

Since d is treated as a constant, the total gate complexity for the full ansatz ($T_{circuit}$) is a polynomial function of n. Consequently, the overall cost of a single VQE iteration scales polynomially with the problem size. This polynomial scaling of per-iteration resources is the primary source of the theoretical advantage of VQE over classical simulation of the quantum process.

However, the total number of iterations, $N_{iter}$, is determined by the classical optimizer’s ability to navigate the high-dimensional energy landscape. This is a non-trivial factor and is not guaranteed to be polynomial. As discussed in Section 4.2 and shown in Figure 7, our empirical analysis for the general trend suggests that $N_{iter}$ may scale exponentially with the number of qubits. This aligns with known challenges for heuristic solvers like VQE, such as the barren plateau phenomenon, where gradients can vanish exponentially.

In summary, our VQE approach possesses a polynomial complexity per iteration. However, due to the potentially exponential scaling of the required optimization iterations, the overall algorithm does not have a proven polynomial time complexity and does not represent a polynomial-time solution to the PTP. Its potential for the quantum advantage is more nuanced. It lies in the possibility that its overall exponential scaling is slower than classical exact solvers, or that it provides higher-quality solutions than fast classical heuristics, as demonstrated in Section 4.2.

## 5. Discussion and Conclusions

The results of our simulation provide a successful proof-of-principle for applying the VQE with a constraint-preserving ansatz to the Profitable Tour Problem. However, this study has several limitations that open avenues for future work.

The primary limitation is the reliance on classical simulation. The exponential time cost of simulation (as shown in the inset of Figure 6) restricts our analysis to small-scale problems, which restricts our ability to demonstrate the potential advantages of executing the algorithm on native quantum hardware. The theoretical promise of such an advantage stems from the polynomial scaling of the quantum circuit’s resources per iteration, a sharp contrast to the exponential resources required for classical simulation. Future work should involve executing the algorithm on real NISQ devices to evaluate its performance under realistic noise and decoherence, which presents a significant engineering challenge.

In addition, the model could be extended to incorporate more realistic tourism constraints. For instance, time windows for visiting specific attractions could be included. While the ansatz construction is a promising step, adapting it to handle such complex, potentially non-local constraints is a non-trivial research question. A possible connection to classical warm-starting techniques, where an initial good solution is used to guide the quantum search, could also be explored to accelerate convergence.

In conclusion, this work demonstrates a promising application of near-term quantum computing to tourism management. The central contribution of this paper is the development and validation of a novel, constraint-preserving variational ansatz specifically tailored for the Profitable Tour Problem. Our methodology directly encodes the problem’s hard constraints into the circuit structure, thereby circumventing the common challenges associated with large penalty terms in the Hamiltonian. Through numerical simulations, we have verified the validity of our VQE-based framework, showing it can find optimal solutions for representative problem instances. Furthermore, our analysis provides a realistic assessment of the algorithm’s complexity, acknowledging that while the resources per iteration scale polynomially, the total number of iterations required for convergence may scale exponentially—a critical challenge for the field of variational algorithms. While the practical application on large-scale problems is currently limited by hardware, this study serves as a crucial proof-of-concept and provides a foundational algorithm for future explorations of quantum optimization in complex, real-world logistics and planning scenarios.

## Figures and Tables

**Table 1:** Some necessary parameters of solving PTP with VQE.

Parameters	Numbers
number of qubits used	nd
number of parameters optimized	nd−d22−d/2
number of terms in Hamiltonian evaluated	$n^{2}d-n^{2}+n$

**Table 2:** An instance of five potential destinations with the associated cost and profits.

Destinations		#1	#2	#3	#4	#5
Cost	the Cartesian coordinates	[−66, 45]	[95, −84]	[−35, −70]	[26, 94]	[15, 20]
Profit	P_1_: Popularity	98	87	92	83	80
	$(W_{1}=0.1$)					
	P_2_: Natural Attractiveness	49	55	97	77	98
	$(W_{2}=0.4$)					
	P3: Cultural Attractiveness	92	89	57	75	40
	($W_{3}=0.02$)					
	P4: Entrance Ticket Price	30	97	56	63	70
	$(W_{4}=-0.18$)					
	P_5_: Crowd Density	30	68	45	83	96
	$(W_{5}=-0.1$)					
	P6: Weather Comfortableness	83	48	100	26	62
	$(W_{6}=0.2$)					
Weighted Average Profit		56.24	66.34	83.72	65.44	82.60

**Table 3:** Success rate of the classical greedy heuristic algorithm across various problem sizes. For each (n,d) combination, the success rate was calculated over 100 randomly generated problem instances. Success is defined as finding the true optimal path, which was determined by a brute-force search.

n	d = 1	d = 2	d = 3	d = 4	d = 5	d = 6
3	82%	59%	50%	-	-	-
4	81%	58%	21%	23%	-	-
5	83%	62%	18%	15%	10%	-
6	81%	57%	30%	19%	11%	4%

## Data Availability

The original contributions presented in this study are included in the article. Further inquiries can be directed to the corresponding author.

## Abbreviations

The following abbreviations are used in this manuscript:

PTP	Profitable Tour Problem
TSP	Traveling Salesman Problem
NISQ	Noisy Intermediate-Scale Quantum Devices
VQE	Variational Quantum Eigensolver
QUBO	Quadratic Unconstrained Binary Optimization
